# Plant Hsp90 Proteins Interact with B-Cells and Stimulate Their Proliferation

**DOI:** 10.1371/journal.pone.0021231

**Published:** 2011-06-20

**Authors:** Mariana G. Corigliano, Andrea Maglioco, Melina Laguía Becher, Alejandra Goldman, Valentina Martín, Sergio O. Angel, Marina Clemente

**Affiliations:** 1 Laboratorio de Biotecnología Vegetal, IIB-INTECH, CONICET-UNSAM, Chascomús, Provincia de Buenos Aires, Argentina; 2 Instituto de Leucemia Experimental (ILEX)-CONICET, Academia Nacional de Medicina, Buenos Aires, Argentina; 3 CESyMA, Escuela de Ciencia y Tecnología, UNSAM, San Martín, Argentina; 4 Laboratorio de Parasitología Molecular, IIB-INTECH, CONICET-UNSAM, Chascomús, Provincia de Buenos Aires, Argentina; University Paris Sud, France

## Abstract

**Background:**

The molecular chaperone heat shock protein 90 (Hsp90) plays an important role in folding stabilization and activation of client proteins. Besides, Hsp90 of mammals and mammalian pathogens displays immunostimulatory properties. Here, we investigated the role of plant-derived Hsp90s as B-cell mitogens by measuring their proliferative responses *in vitro*.

**Methodology:**

Plant cytosolic Hsp90 isoforms from *Arabidopsis thaliana (*AtHsp81.2) and *Nicotiana benthamiana* (NbHsp90.3) were expressed in *E. coli*. Over-expression of recombinant plant Hsp90s (rpHsp90s) was confirmed by SDS-PAGE and western blot using and anti-AtHsp81.2 polyclonal anti-body. Both recombinant proteins were purified by Ni-NTA affinity chromatography and their identity confirmed by MALDI-TOF-TOF. Recombinant AtHsp81.2 and NbHsp90.3 proteins induced prominent proliferative responses in spleen cells form BALB/c mice. Polymyxin-B, a potent inhibitor of lipopolysaccharide (LPS), did not eliminate the rpHsp90-induced proliferation. In addition, *in vitro* incubation of spleen cells with rpHsp90 led to the expansion of CD19-bearing populations, suggesting a direct effect of these proteins on B lymphocytes. This effect was confirmed by immunofluorescence analysis, where a direct binding of rpHsp90 to B- but not to T-cells was observed in cells from BALB/c and C3H/HeN mice. Finally, we examined the involvement of Toll Like Receptor 4 (TLR4) molecules in the rpHsp90s induction of B-cell proliferation. Spleen cells from C3H/HeJ mice, which carry a point mutation in the cytoplasmic region of TLR4, responded poorly to prAtHsp90. However, the interaction between rpHsp90 and B-cells from C3H/HeJ mice was not altered, suggesting that the mutation on TLR4 would be affecting the signal cascade but not the rpHsp90-TLR4 receptor interaction.

**Conclusions:**

Our results show for the first time that spleen cell proliferation can be stimulated by a non-pathogen-derived Hsp90. Furthermore, our data provide a new example of a non-pathogen-derived ligand for TLRs.

## Introduction

The 90-kDa heat shock proteins (Hsp90s) belongs to a widespread family of molecular chaperones found in bacteria and all eukaryotes. Many eukaryotes possess multiple Hsp90 homologs, including endoplasmic reticulum- mitochondrial- and chloroplast-specific isoforms [1]. Hsp90s function as general chaperones, playing important roles in many essential cell functions as a result of their molecular chaperonin features: protein translocation, folding and assembly [2], [3], [4]. This is due to the interaction of Hsp90 with various proteins (client proteins) with a different degree of specificity, which leads to modulating their conformation. Its clients include proteins as structurally and functionally different as telomerases, polymerases, kinases and a range of nuclear hormone receptors [1], [5], [6], [7].

Noteworthy, Hsp90 has also been found to have a specific function in immunological processes. An increasing body of data suggests that certain Hsp90s play a role in both innate and adaptive immunity, and in some cases, the adjuvant effect of Hsp90s have been assessed [8], [9], [10], [11], [12], [13]. Hsp90s can elicit potent specific cellular adaptive immune responses based on their ability to chaperone antigenic peptides, and also act independently of chaperoned peptides to directly stimulate innate immune responses [14], [15], [16], [17]. Given the ancient origin of Hsp90s, such specialization may have occurred early in evolution and, therefore, it is feasible that these immunological properties of Hsp90 from humans and other organisms like bacteria and parasites are also present in their plant orthologs. In fact, plant Hsp90s are able to interact with animal co-chaperones and cooperate with them in the folding process, suggesting plasticity between chaperone complexes from different eukaryotic organisms [3], [18], [19]. An open question is whether plant Hsp90s also present immunostimulatory properties as those observed in animal and protozoan Hsp90s. This is of importance because plants are considered novel bioreactors to produce pharmaceutical and vaccine molecules [20]. However, since the production of high amounts of antigen in plants is generally difficult, there is a need to develop different strategies [21], [22], [23]. An interesting option is to express the polypeptide of interest in plants with a carrier that could provide stability and therefore increase the polypeptide production [24]. Should Hsp90s from plants present adjuvant properties, they could arise as novel and interesting carriers for proteins or peptides of immunoprotective value, improving the immunogenicity property of the transgenic plant extract.

In the present work, we evaluated the ability of recombinant *Nicotiana benthamiana* and *Arabidopsis thaliana* Hsp90s to induce *in vitro* proliferation of splenocytes from naïve BALB/c, C3H/HeN and C3H/HeJ mice. In addition, we determined which subpopulations of spleen cells were stimulated by recombinant plant Hsp90s using flow cytometry. Our data indicate that their proliferative capacity is related to the fact that plant Hsp90s behave as potent B-cell mitogens. On the other hand, we showed by immunofluorescence analysis that rAtHsp81.2 co-localizes with anti-CD19 but not with anti-CD3 labeling, suggesting that rAtHsp81.2 interacts specifically with B-cells on their surface.

## Results

### Isolation of the *A. thaliana* and *N. benthamiana* Hsp90 coding region

**Figure 1:**
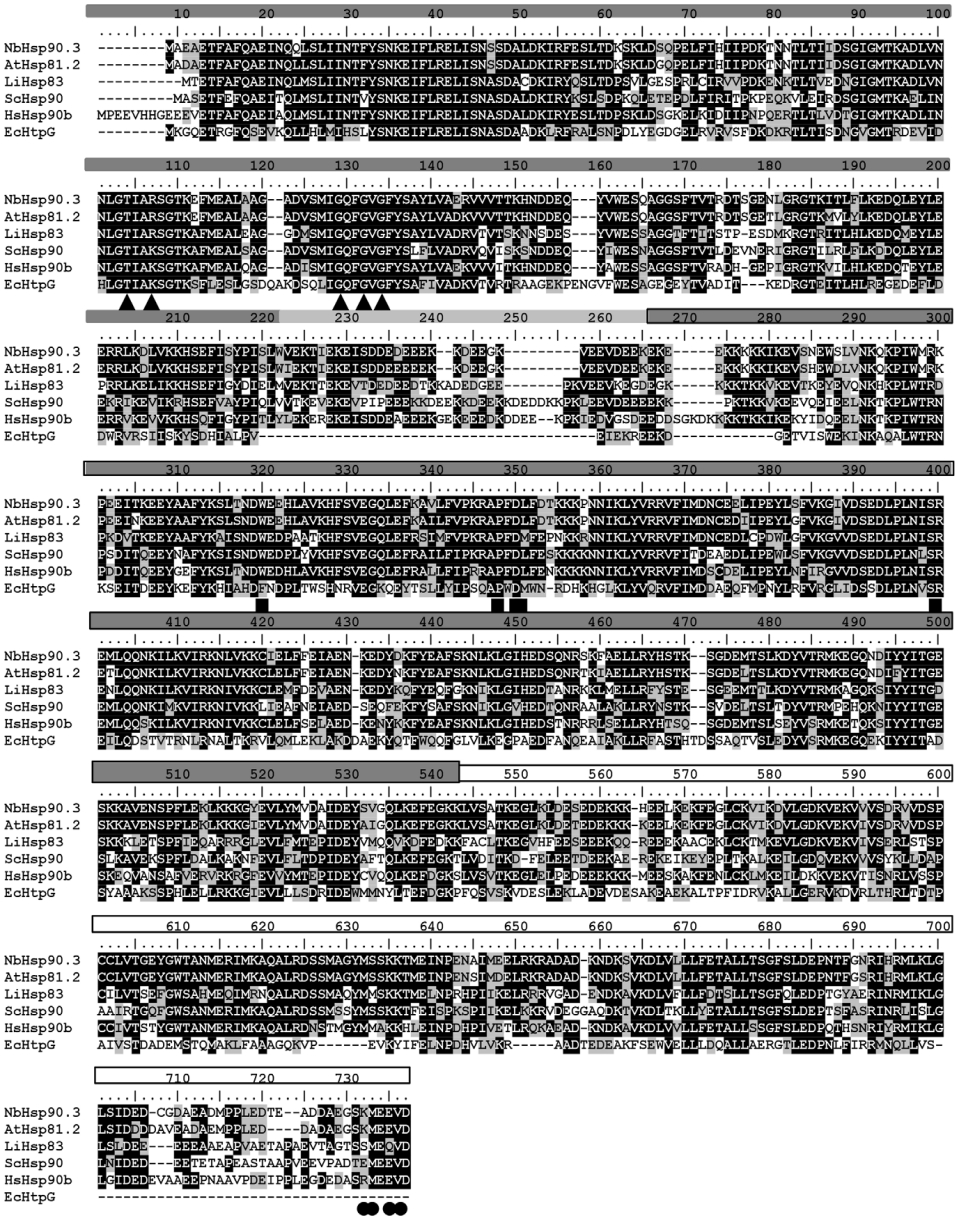
Multiple sequence alignment of Hsp90 proteins from *N. benthamiana, A. thaliana, L. infantum, S. cerevisiae, H. sapiens* and *E. coli.* The different colors of the bar under the alignment represent different domains of protein as follows: N-terminal domain; Linker region; middle domain region; and C-terminal domain. The different structures on the bar represents functionally important residues i.e. ▴ATP binding site; ▪ Client binding site and • Co-chaperone binding site.

The *A. thaliana* Hsp81.2 (AtHsp81.2) open reading fame (ORF) was successfully amplified from the plasmid RAFL 09-06-O18 (provided by RIKEN BRC) using PCR, whereas the *N. benthamiana* Hsp90.3 (NbHsp90.3) ORF (accession N° GQ_845021) was amplified from cDNA by PCR. Both coding regions were inserted into pRSET-A. The amino acid sequences of AtHsp81.2 and NbHsp90.3 were compared to those of other eukaryotic Hsp90 proteins (Fig.1). The multiple sequence alignment (MSA) revealed a 93.4% amino acid sequence identity between AtHsp81.2 and NbHsp90.3. Besides, the analysis of the MSA revealed that the sequences of plant Hsp90 share a high similarity with *Leishmania infantum* Hsp83 and *Homo sapiens* Hsp90b (around 65% and 68% amino acid sequence identity, respectively). As previously reported [1], the crucial residues for ATP binding are highly conserved in eukaryotic and prokaryotic Hsp90s, while the residues involved in the client and co-chaperone binding sites are highly conserved in human, yeast, parasitic and plant Hsp90 but not in bacteria (Fig. 1).

### Expression and purification of *A. thaliana* and *N. benthamiana* Hsp90 proteins

**Figure 2:**
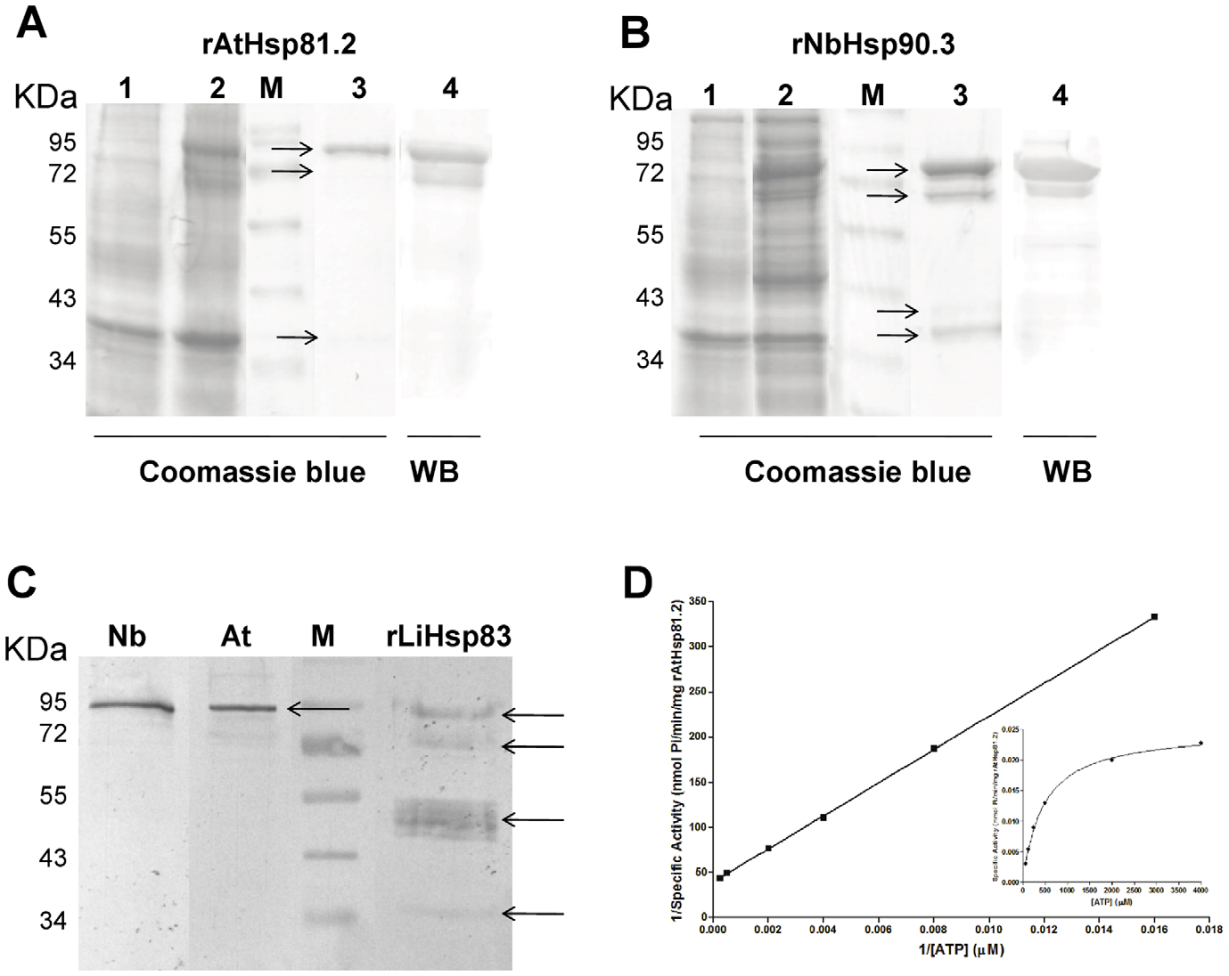
Analysis of the expression of plant Hsp90. (A) Expression and purification of recombinant *A. thaliana* Hsp81.2. (B) Expression and purification of recombinant *N. benthamiana* Hsp90.3. The expression and purification of the recombinant proteins were checked by Coomassie blue-stained polyacrylamide gel and western blot using an anti-rAtHsp81.2. Lane M: protein mass markers; lane 1: lysate of *E. coli* Rosetta (DE3) without induction; lane 2: lysate of *E. coli* Rosetta (DE3) after IPTG induction; lane 3: elute after Ni^+^ affinity purification; Lane 4: western blot from elute after Ni^+^ affinity purification using a polyclonal antibody anti-rAtHsp81.2. Arrows indicate the bands that were sent to analyze by MALDI-TOF-TOF. (C) Western blot analysis, where the anti-AtHsp81.2 was able to recognize the recombinant LiHsp83 protein and the Hsp90 proteins from plant extract. Nb: protein extracts from *N. benthamiana* leaves; At: protein extracts from *A. thaliana* leaves; rLiHsp83: recombinant LiHsp83 expressed and purified from *E. coli*. M: protein mass markers. Arrows indicate the bands detected by anti-AtHsp81.2. (D) The ATPase activity of rAtHsp81.2 was determined using varying concentrations of ATP (0–3000 µM). The amount of phosphate released was determined colorimetrically. The kinetic parameters *K_M_* and *V_max_* were determined using a Lineweaver–Burke plot. The inset shows the Michaelis–Menten plot generated with the data. All points are the means triplicate samples of three independent experiments.

Each expression plasmid was transformed into *E. coli* Rosetta (DE3) pLys S for protein expression. The recombinant AtHsp81.2 and NbHsp90.3 (rAtHsp81.2 and rNbHsp90.3) were soluble and migrated with an apparent molecular mass of 80 kDa (lane 2, Fig. 2A and B). Since recombinant plant Hsp90s (rpHsp90s) were fused to the His-Tag, rNbHsp90 and rAtHsp81.2 were conveniently purified with the Ni-NTA agarose column, yielding proteins of ∼80 kDa (lane 3, Fig. 2A and B). The identity of rAtHsp81.2 and rNbHsp90.3 was confirmed by mass spectrometry. Some additional bands were also observed and the mass spectrometry analysis confirmed that they were proteolytic products of their respective N-termini. A polyclonal antiserum against rAtHsp81.2 was raised in mice. By immunoblotting the serum recognized a strong band corresponding to rAtHsp81.2 and rNbHsp90.3, as well as some other weaker ones (lane 4, Fig. 2A and B). This antiserum was able to recognize the recombinant LiHsp83 protein [9] and the Hsp90 proteins from plant extract (Fig. 2C). The high protein yields obtained after affinity purification facilitated the biochemical characterization of the basal rpHsp90 ATPase activity. Michaelis-Menten constant (*K_m_*) and maximum velocities (*V_max_*) for ATP were determined by the malachite green assay [25]. The ATPase activity for rpHsp90 was assessed by the detection of phosphate released from the cleavage of ATP. rAtHsp81.2 was shown to have a basal ATPase activity with a *V_max_* of 0.02497 nmol of phosphate released per minute per milligram of rAtHsp81.2 and a *K_m_* of 461 µM (Fig. 2D). *K_m_* and *V_max_* for rNbHsp90.3 were 683 µM and 46.6 nmol of phosphate released per minute per milligram of rNbHsp90.3, respectively (data not shown). This analysis revealed that the recombinant proteins purified from bacteria maintain their ATPase activity, indicating the purification of active forms of the chaperones.

### Splenocytes from naïve BALB/c mice proliferate in response to *A. thaliana* and *N. benthamiana* Hsp90 proteins

**Figure 3:**
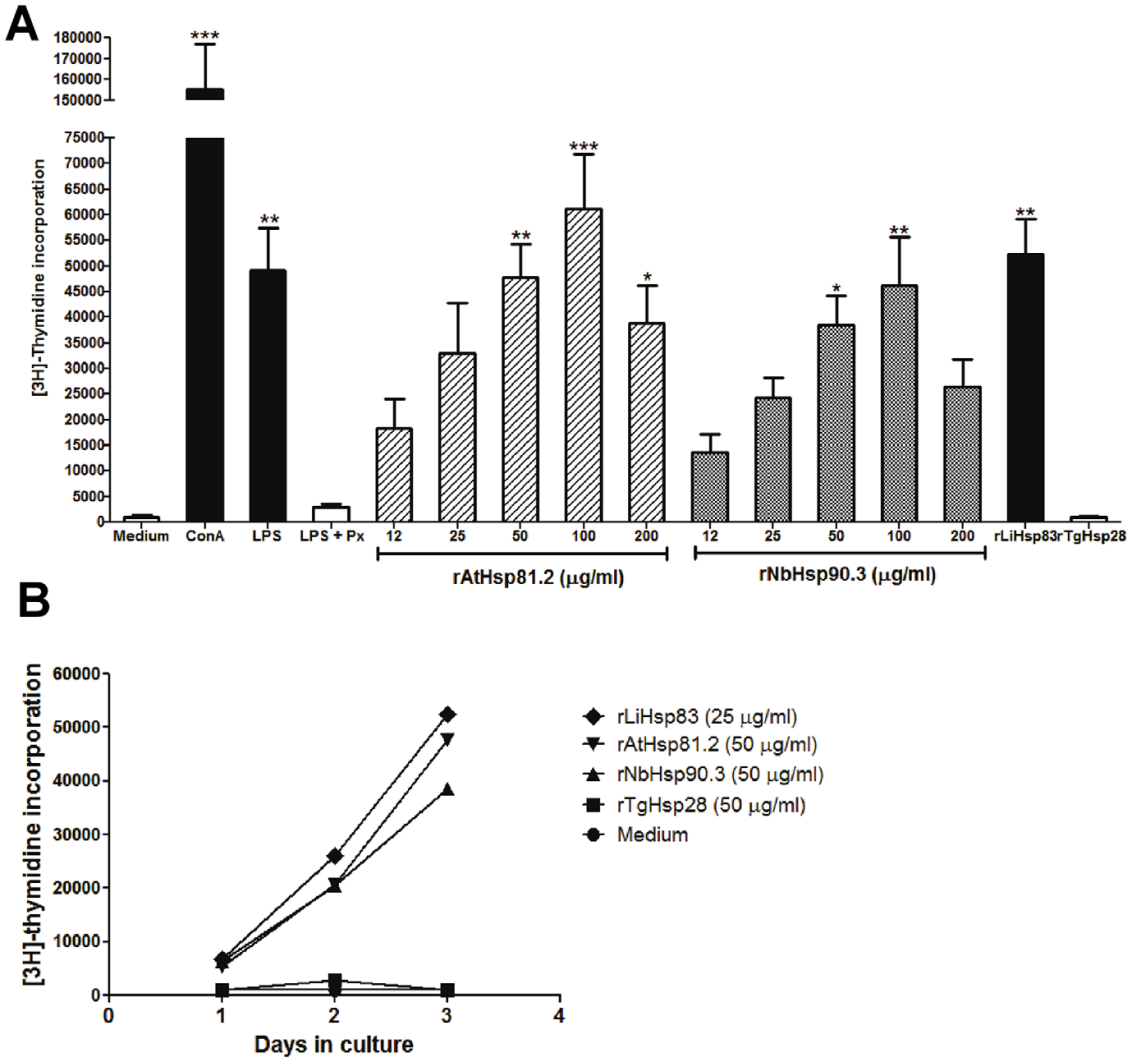
The stimulation ability of rpHsp90 in spleen cells from BALB/c mice. (A) Proliferative responses of splenocytes to various stimuli. The splenocytes were incubated for 72 h at 37°C in 5% CO_2_. (B) Kinetics of splenocyte proliferations. The concentrations of the different stimuli were: ConA 5 µg/ml, LPS 10 µg/ml, rLiHsp83 25 µg/ml and rTgHsp28 50 µg/ml. Polymyxin B (PX) at 2 µg/ml was added to the wells containing the recombinant Hsps proteins and LPS. Values represent the mean counts per minute and standard deviations of triplicate samples and are representative of three experiments. ConA: concanavalin A; LPS: lipopolysaccharides; rAtHsp81.2: recombinant heat shock protein 81.2 from *Arabidopsis thaliana*; rNbHsp90.3: recombinant heat shock protein 90.2 from *Nicotiana benthamiana*; rLiHsp83: recombinant heat shock protein 83 from *Leishmania infantum*; rTgHsp28: recombinant small heat shock protein 28 from *Toxoplasma gondii*. The asterisks indicate the statistically significant differences between the different stimuli and the negative controls (medium, LPS + PX and rTgHsp28). Statistical analysis was performed by one-way analysis of variance (ANOVA) using the Bonferroni's Multiple Comparison Test. *p<0.05, **p<0.01, ***p<0.001.

We next examined the ability of rpHsp90s to stimulate spleen cells from BALB/c mice. Stimulation abilities of *L. infantum* rLiHsp83 [9] and *Toxoplasma gondii* small heat shock protein rTgHsp28 chaperone [26] were tested as controls. rpHsp90s and rLiHsp83 induced prominent proliferative responses of spleen cells in the presence of polymyxin-B (PX), whereas rTgHsp28 did not induce this proliferative response (Fig. 3A). Therefore, this proliferative response of spleen cells was specifically induced by rpHsp90 and rLiHsp83. rpHsp90-induced proliferative responses were observed when concentrations as low as 12 µg/ml were used and plated at 100 µg/ml (Fig. 3A). Since significant differences were observed when concentrations of rpHsp90 were equal to or higher than 50 µg/ml, the following experiments were carried out using 50 µg/ml of recombinant proteins.

The proliferative capacity of LPS and ConA was also evaluated. As expected, both LPS and ConA were able to induce proliferative responses of spleen cells. PX treatment was able to abolish LPS proliferative capacity but not the rpHsp90 one, suggesting that the proliferative capacity of the rAtHsp81.2 and rNbHsp90.3 preparations must be attributed to the proteins and not to LPS contamination (Fig. 3A and Fig. S1).

In the next experiment, a time course was determined after stimulation with rpHsp90s. After splenocyte incubation (2.5×10^5^ cells/well) with rAtHsp81.2 and rNbHsp90.3, [^3^H]-thymidine incorporation was increased in a time-dependent manner only when rpHsp90s and rLiHsp83 were present (Fig. 3B). The kinetics of [^3^H]-thymidine incorporation in splenocytes stimulated with rpHsp90s and rLiHsp83 were similar. None of the controls (medium or rTgHsp28) showed any [^3^H]-thymidine incorporation in the cultures during the time course assayed (Fig. 3B).

### *A. thaliana* and *N. benthamiana* Hsp90 induced proliferation of B-cells but not of T-cells

**Figure 4:**
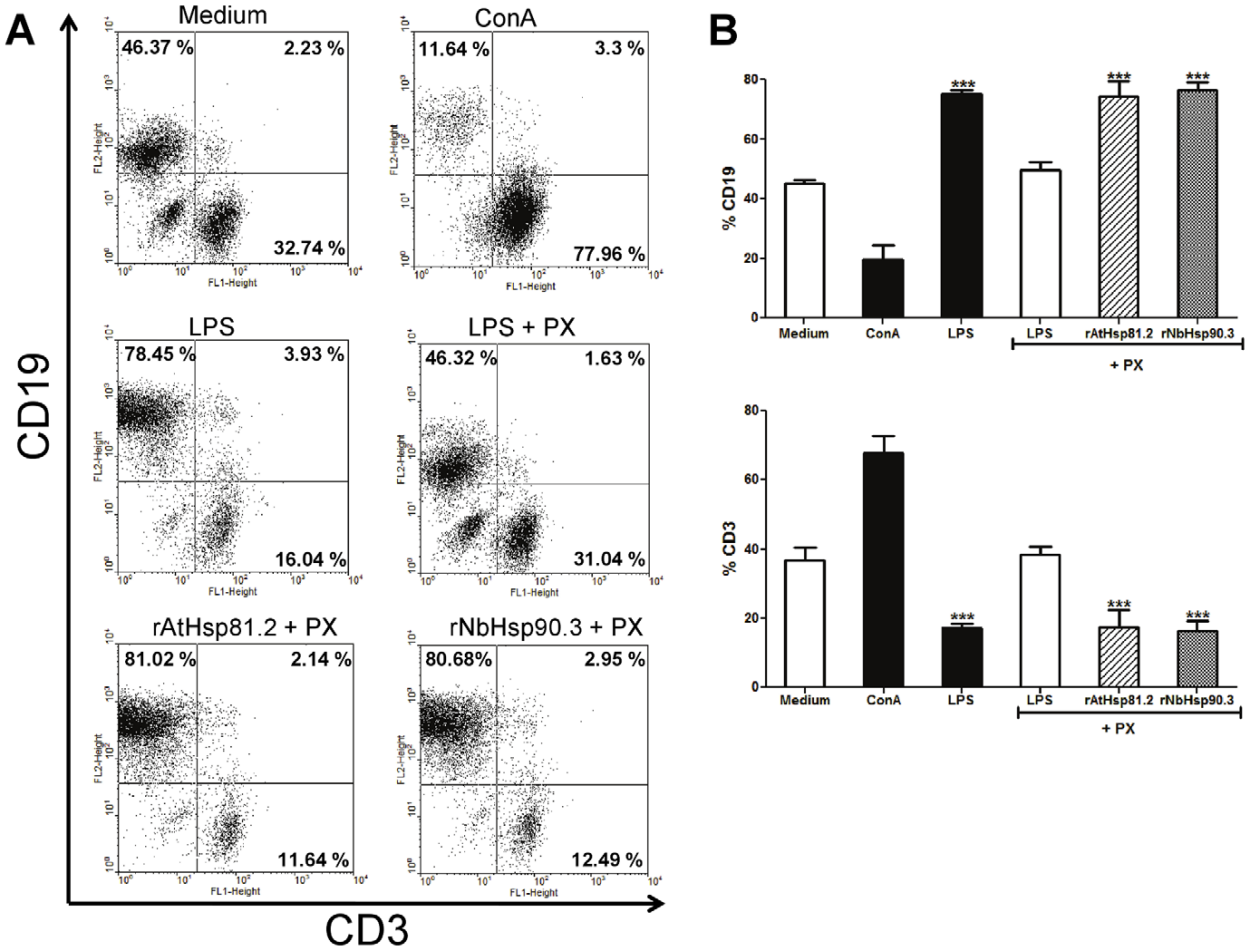
Determination of B-cell and T-cell populations in splenocyte cultures after stimulation with the different stimuli. (A) After incubation for 3 days with ConA 5 µg/ml, LPS 10 µg/ml, LPS 10 µg/ml + PX, rAtHsp81.2 50 µg/ml + PX and rNbHsp90.3 50 µg/ml + PX, cells were incubated with either anti-CD3 (T cell) or anti-CD19 (B cell) and analyzed by flow cytometry to determine the percentage of T and B cells. Polymyxin B (PX) was used at 2 µg/ml. (B) Percentages of CD3 and CD19 after each stimulus were graphicated. ***p<0.001 LPS, rAtHsp81.2, rNbHsp90.3 vs. medium, ConA and LPS + PX. Values represent the mean and standard deviations of triplicate samples of three independent experiments. Statistical analysis was performed by one-way analysis of variance (ANOVA) using the Bonferroni's Multiple Comparison Test.

B- and T-lymphocytes are the most abundant cells present in the murine spleen, accounting for about 90% of the total number of cells [11]. To identify the subset of cells that was proliferating in response to rpHsp90, splenocytes from BALB/c mice were labeled with fluorophore-conjugated antibodies against T- or B-cell specific markers (CD3 and CD19 receptor, respectively) after 3 days of stimulation (Fig. 4). The percentage of no stimulated splenocytes, we determined that in average 45.3% of the cells were CD19 positive and that 36.8% were CD3 positive. A significant increase in CD3 cells (accounting for 77.9%) was observed in ConA-stimulated splenocytes, as expected for a T-cell mitogen. In contrast, after LPS stimulation, the percentage of CD19 cells significantly increased to 78.4%, as expected for a B-cell specific mitogen. The addition of PX in the LPS preparation significantly modified the total number of CD19 and CD3 cells compared to LPS alone (Fig. 4A and B). Interestingly, stimulation of splenocytes with rAtHsp81.2 and rNbHsp90.3 plus PX promoted a marked proliferation of CD19 B cells (81% and 80.6%, respectively), while CD3 T-cells did not respond to rpHsp90 (Fig. 4A and B). Thus, the results suggest that the main spleen cell population proliferating in response to rpHsp90s is constituted by B-lymphocytes.

**Figure 5:**
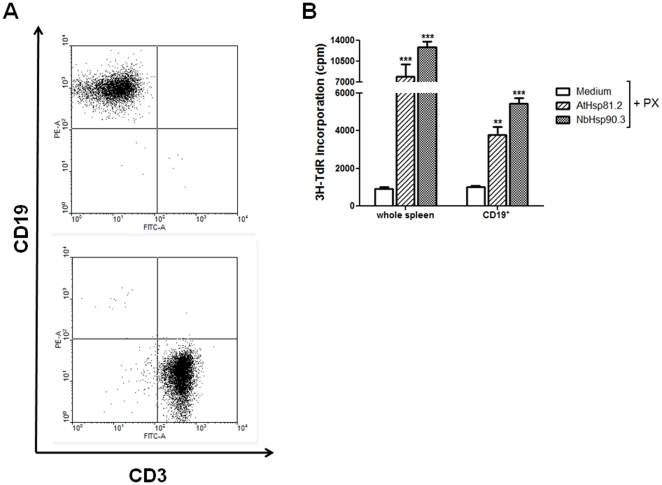
rpHsp90-induced proliferation of B-cell population. (A) Fractionation of spleen cells. Whole spleen cells from BALB/c mice were incubated with (PE)-conjugated anti-mouse CD19 and FITC–conjugated anti-mouse CD3. CD19^+^ and CD3^+^ populations were fractionated by a positive sorting. (B) 2.5×10^5^ whole spleen cells and 1×10^5^ fractionated CD19^+^ cells were cultured for 3 days with or without rAtHsp81.2 or rNbHsp90.3. Polymyxin B (PX) was used at 2 µg/ml. Values represent the mean and standard deviations of triplicate samples and are representative of two experiments. Statistical analysis was performed by one-way analysis of variance (ANOVA) using the Bonferroni's Multiple Comparison Test. The asterisks indicate the statistically significant differences between the different stimuli and medium. **p<0.01, ***p<0.001.

To confirm that B-lymphocytes were the cell type responsible for the proliferation observed after incubation with rpHsp90, spleen cells were subfractionated in CD19 and CD3 populations by a positive sorting. The purity of CD19 and CD3 populations was 96% and 93%, respectively (Fig. 5A). These fractionated cells were cultured with or without rpHsp90. Stimulation with rpHsp90 induced a marked proliferation of CD19 B-cells (Fig. 5B), while the CD3 population was not able to be measured because [^3^H]-thymidine levels were too low. Interestingly fractionated B cell (CD19^+^) showed lower stimulation rates than those observed from whole spleen cells (Fig. 5B).

### B-cells from Toll-Like Receptor 4-Defective C3H/HeJ Mice did not respond to *A. thaliana* and *N. bentamiana* Hsp90-stimulus

**Figure 6:**
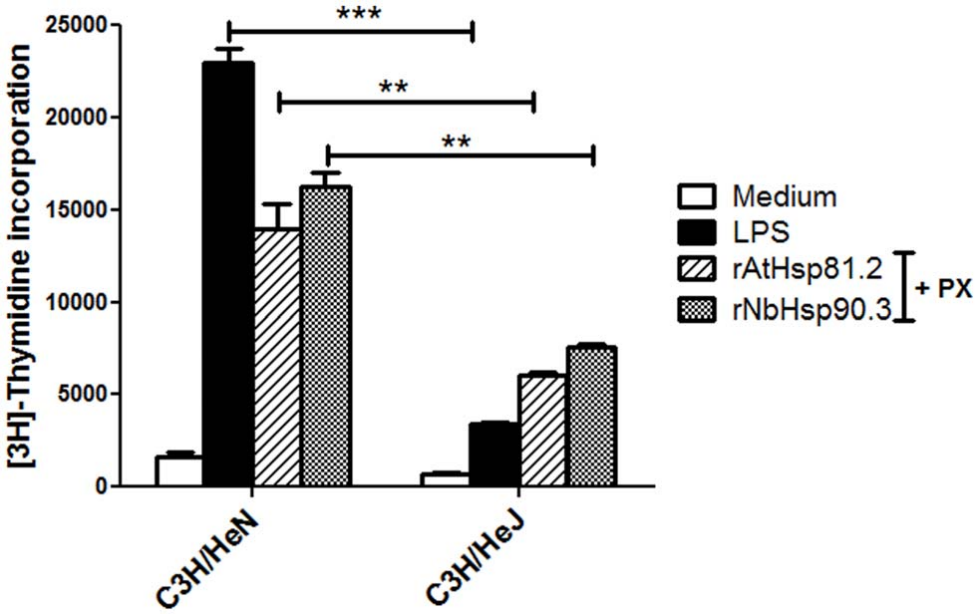
Involvement of TLR4 molecules in recombinant plant Hsp90-induced spleen cell proliferation. rAtHsp81.2, rNbHsp90.3 or LPS–induced proliferative responses of 2.5×10^5^ spleen cells from C3H/HeJ mice were compared with those of C3H/HeN mice. The concentrations of the different stimuli were: LPS 10 µg/ml, rAtHsp81.2 50 µg/ml and rNbHsp90.3 50 µg/ml. PX at 2 µg/ml was added to the wells containing the recombinant Hsps proteins. Statistical analysis was performed by two-way analysis of variance (ANOVA) using the Bonferroni's Post-Test. *p<0.05, **p<0.01, ***p<0.001. Values represent the mean and standard deviations of triplicate samples and are representative of two experiments.

It has been previously observed that Hsp90 can interact with the toll-like receptor 4 (TLR4) [27]. C3H/HeJ mice possess a spontaneous mutation in the TLR4 gene (*Tlr4Lps-d*) that results in a mutation in the cytosolic region of the protein. Hence, these C3H/HeJ mice are more resistant to endotoxin and display a lower response to LPS treatment (http://jaxmice.jax.org/strain/000659.html). By using splenocytes from C3H/HeJ mice, we examined the involvement of TLR4 in the rpHsp90 induction of B-cell proliferation. Spleen cells from C3H/HeJ mice responded poorly to rAtHsp81.2, rNbHsp90.3 or LPS. Spleen cells from C3H/HeN mice proliferated as well as those from BALB/c mice (Fig. 6), indicating that rpHsp90 B-cell mitogenic properties would involve the TLR4 receptor.

### Direct binding of *A. thaliana* Hsp90 to B-cells

**Figure 7:**
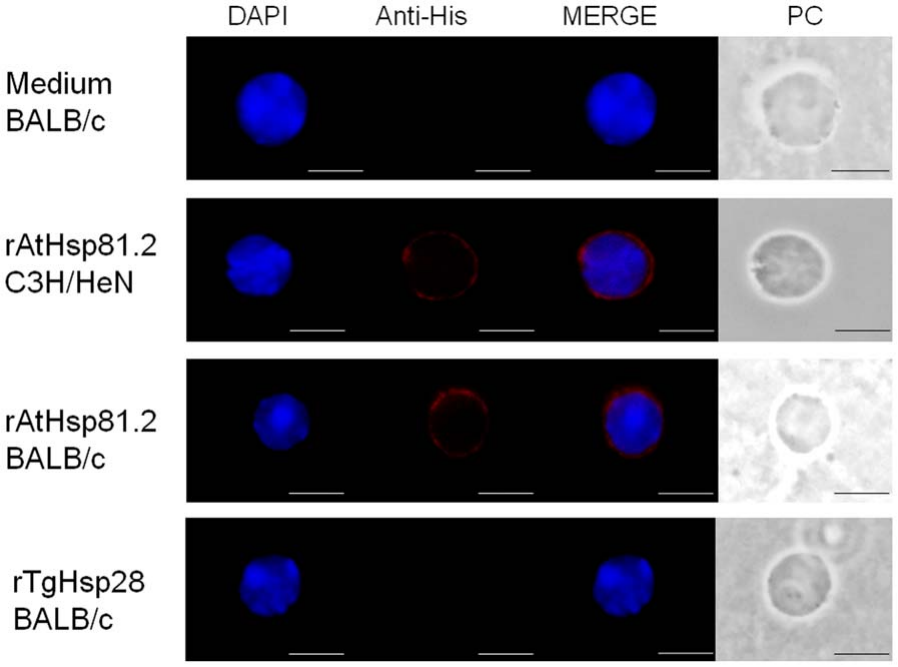
Recombinant AtHsp81.2 localization at the plasma membrane from spleen cells. Spleen cells were stimulated with AtHsp81.2, TgHsp28 or medium for 30 min. rAtHsp81.2 was incubated with anti-His and Alexa Fluor 594 goat anti-mouse IgG (Invitrogen, red color) was used as secondary antibody. Nuclei were stained with DAPI. Both images were merged (rAtHsp81.2+ nucleus). Scale bar represents 1 µm. This image is representative of a larger field of view, and data are from a representative experiment performed three times. PC: phase contrast.

**Figure 8:**
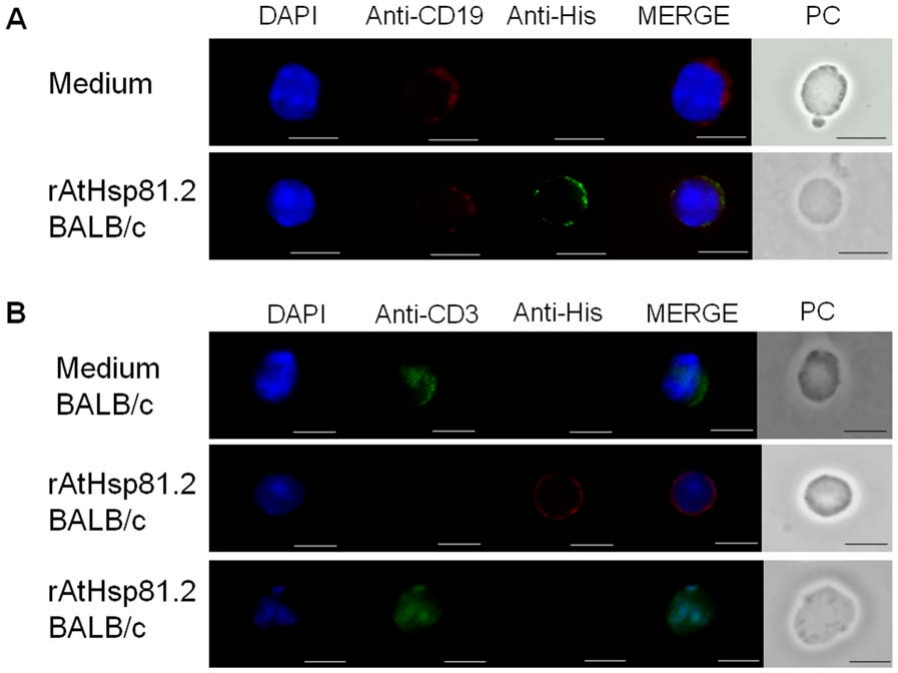
AtHsp81.2 at the plasma membrane co-localizes with CD19 cells but not with CD3 cells. Spleen cells were stimulated with either rAtHsp81.2 or medium for 30 min. (A) CD19^+^ cells were incubated with mouse anti-6His mAb as primary antibody, Alexa Fluor 488 goat anti-mouse IgG (green color) as secondary antibody and phycoerythrin (PE)-conjugated anti-mouse CD19 mAb (BD, red color) as tertiary antibody. Nuclei were stained with DAPI. The three images were merged (CD19+ rAtHsp81.2+ nucleus). (B) CD3^+^ cells were incubated with mouse anti-6His mAb as primary antibody, Alexa Fluor 594 goat anti-mouse IgG (red color) as secondary antibody and fluorescein isothiocyanate (FITC)-conjugated anti-mouse CD3 mAb (BD, green color). Nuclei were stained with DAPI. The three images were merged (CD3+ rAtHsp81.2+ nucleus). Scale bar represents 1 µm. This image is representative of a larger field of view, and data are from a representative experiment performed three times. PC: phase contrast. Green, red and blue fluorescence were recorded separately, and the images were merged using image-pro plus 4.5.

**Figure 9:**
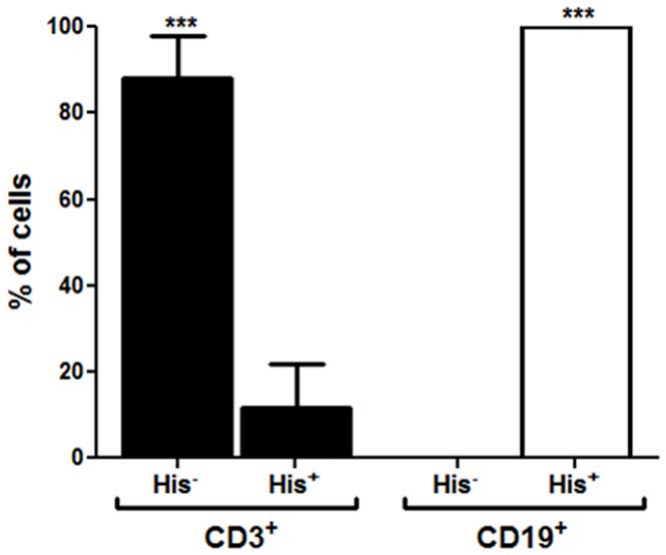
Percentage of CD3 and CD19 cells that co-localize with rAtHsp81.2 protein. Spleen cells were stimulated with either rAtHsp81.2 or medium for 30 min and incubated with mouse anti-6His mAb as primary antibody, Alexa Fluor 488 goat anti-mouse IgG (green color) as secondary antibody and phycoerythrin (PE)-conjugated anti-mouse CD19 mAb (BD, red color). For detection of CD3^+^ lymphocytes, the cells were incubated with mouse anti-6His mAb as primary antibody, Alexa Fluor 594 goat antimouse IgG (red color) as secondary antibody and fluorescein isothiocyanate (FITC)-conjugated anti-mouse CD3 mAb (BD, green color). Nuclei were stained with DAPI. The three images were merged (CD19+ rAtHsp81.2+ nucleus, or CD3 + rAtHsp81.2 + nucleus).To calculate the percentage values, two hundred CD3^+^ and one hundred CD19^+^ cells were analyzed from two independent experiments. Statistical analysis was performed by two-way analysis of variance (ANOVA) using the Bonferroni's Post-Test. CD3^+^/His^-^ vs. CD3^+^/His^+^ and CD19^+^/His^−^ vs. CD19^+^/His^+^: ***p<0.001.

In order to determine whether the B-cell proliferation effect observed by prHs90 involves a direct binding of rpHsp90 to B-cells, an immunofluorescence analysis was performed. Splenocytes from BALB/c mice were isolated, incubated with rAtHsp81.2 or rNbHsp90.3, fixed and immunolabeled with anti- rAtHsp81.2 or anti-His and anti-CD3 or ant-CD19 antibodies. Since anti-rAtHsp81.2 produced certain level of background (data not shown), the anti-His antibody was chosen for co-localization analysis. It was observed that the anti-His antibody localized at the plasma membrane only when the cells were incubated with rAtHsp81.2 but not with TgHsp28 -another recombinant protein with a 6-His tag- or medium alone (Fig. 7). Besides, the presence of rAtHsp81.2 at the plasma membrane co-localized with the labeling of anti-CD19 antibody but not with that of anti-CD3 antibody (Fig. 8A and B, Fig. S2). This result is supported with a statistical analysis where all labeled CD19 cells co-localized with the His^+^ cells (100%), while only a few labeled CD3 cells were also labeled with anti-His antibody (15%) (Fig. 9).

Since B-cells from C3H/HeJ mice were unable to proliferate in the presence of rpHsp90s, but expressed the TLR4 receptor, we tested the ability of rpHsp90s to interact with these cells. Splenocytes from C3H/HeJ mice were treated as described above and showed that rAtHsp81.2 is present on the surface of B-cells (CD19 positive), indicating that the inability of these B-cells to proliferate when incubated with rpHsp90 is due to TLR4 based-signaling impairment rather than rpHsp90-B cell interaction (Fig. S3).

## Discussion

Recently, Heat shock proteins such as Hsp60, Hsp70, Hsp90 and gp96 have been reported to play important roles in antigen presentation, activation of lymphocytes and macrophages, and activation and maturation of dendritic cells (APCs) [28], [29], [30], [31], [32], [33], [34]. Here we demonstrated that plant Hsp90s function as B-cell mitogen. The kinetic patterns of plant Hsp90-induced proliferative responses of spleen cells showed that 50 µg/ml of the recombinant protein is sufficient to induce a significantly proliferative response in spleen cells from naïve BALB/c and C3H/HeN mice. Flow cytometry analysis of the responder cells revealed that rpHsp90s induced proliferation of B- but not of T-cells, indicating that plant Hsp90s possesses B-cell mitogenic properties. In this work, rLiHsp83 was used as a positive control because its effect on B-cells has been previously reported [9], [11], showing that rpHsp90s and rLiHsp83 share similar immunostimulatory properties. Our results show for the first time how spleen cell proliferation can be stimulated by non-pathogen-derived Hsp90s.

The amino acid sequences from plant Hsp90 showed high identity with its ortholog from humans and other eukaryotic organisms, supporting the idea that its function in cells is highly conserved in eukaryotes. In fact, Hsp90 purified from *Brassica napus* (BnHsp90) was able to bind to Hsp70/Hsp40 from rabbits and to Hop and p23 from humans [3], [18]. Following this line of thinking, and taking into account the high degree of sequence conservation between the plant and the parasitic Hsp90 (65% of identity), it is logical that eukaryotic Hsp90s share similar functions, including their immunostimulatory properties. This is similar to that observed for Hsp70, a highly conserved chaperone among eukaryotic organisms. It has been recently demonstrated that plant-derived Hsp70 shares structural and functional properties with the mammalian homolog, suggesting that Hsp70 could be an available immunological carrier [35]. In addition, Hsp70s from *T. gondii*, *L. infantum* and *Mycobacterium tuberculosis* have been demonstrated to be potent mitogens for murine splenocytes [11], [36], [37].

Because LPS is a well-known B-cell mitogen, *a priori* it cannot be discarded that the splenocyte proliferation observed by rpHsp90s was due to LPS contamination. However, rpHsp90-induced proliferative responses were not significantly inhibited by polymyxin-B, which almost completely inhibited LPS-induced proliferative responses. On the other hand, another heat shock protein, rTgHsp28 purified by the same method as rpHsp90, was unable to induce proliferation, suggesting that the low contamination with LPS in the samples were inhibited by polymyxin-B. In addition, the absence of proliferation in splenocytes stimulated with rTgHsp28, a protein with intact chaperone activity [26], suggests that other contaminations such as lipoproteins and flagellins, which may also be non-specifically chaperoned in the recombinant Hsp products, have little chances to be responsible for the splenocyte proliferation observed in response to the recombinant plant Hsp90 preparations.

Immunostimulation by Hsp60, Hsp70 and gp96 is thought to be mediated by members of the conserved TLR family. In particular, TLR2 and TLR4 have been identified to be responsible for Hsp60- and Hsp70- as well as for gp96-induced APC activation [38]. Recent accumulating evidence indicates that TLR signaling pathways consist, at least, of a MyD88-dependent pathway that is common to all TLRs, and a MyD88-independent pathway that is specific of the TLR3 and TLR4 signaling pathways [39], [40]. The Gp96-TLR2/4 interaction results in activation of nuclear factor B and mitogen- and stress-activated protein kinases [27]. In addition, in TLR2^−/−^ mice, the lack of TLR2 signaling can apparently be completely compensated by TLR4, but TLR2 can induce only minimal activation when TLR4 is not functional [27]. On the other hand, *T. gondii* TgHsp70-induced proliferative responses of spleen cells required TLR4 molecules as receptors, while TgHsp70 induced proliferative responses in spleen cells of MyD88^−/−^ mice, suggesting the participation of a MyD88-independent pathway downstream of TLR4 in TgHsp70-induced proliferative responses [36]. The fact that rpHsp90s had no effect on B-cells from C3H/HeJ mice suggests that the action of rpHsp90 on B-cells from BALB/c or C3H/HeN mice should be related to TLR4 as it was observed by its endoplasmic reticulum counterpart (gp96) [27]. It remains to be determined whether the rpHsp90 pathway is completely independent of TLR2 and MyD88, as observed in Gp96 and TgHsp70.

According to our results, rpHsp90 would be stimulating lymphocyte B proliferation mainly through the cell surface receptor TLR4. This finding is fully supported by the labeling of the B-cell with rpHsp90. This is the first demonstration of a specific localization of extracellular and heterologous Hsp90 on the B-cell surface. Among differences on receptor repertoire between B- and T-cells are TLR4, TLR2 and CD19, all of them present in B-cells but not T-cells. Therefore, interaction between rpHsp90 and B-cells could be through one or some of these receptors. TLR4 and TLR2, but not CD19, are related with lymphocyte proliferation [36]. Since B-cells with a mutation in TLR4 were unable to proliferate in the presence of rpHsp90, it could be considered that TLR4 is at least one of the receptors involved in the interaction. Our results suggest that plant Hsp90s interact with the mutant TLR4 receptor, suggesting that the mutation on TLR4 would be affecting the signal cascade but not the rpHsp90-TLR4 receptor interaction. These data provide a new example of a non-pathogen-derived ligand of TLRs.

An interesting observation is that the proliferation of the highly purified B cells is significantly lower than that obtained with the whole spleen cell population, suggesting that this proliferation could also be assisted by accessory cells. In fact, several reports demonstrated that some Hsps bind to the surface of professional antigen-presenting cells (APC) and are internalized spontaneously by receptor-mediated endocytosis demonstrating the existence of specific receptors for Hsp on professional APC [41]–[43].

The main advantage of plant expression systems over other vaccine production systems is the reduced manufacturing cost [44]. Over the past two decades, vaccine antigens expressed via the plant nuclear genome have elicited appropriate immunoglobulin responses and have conferred protection upon oral delivery [45]. However, stably integrated nuclear transgenes typically yield relatively low levels of expression (<1% total soluble protein, TSP). In consequence, several strategies have been developed to increase the levels of recombinant proteins for plant production systems [44]. Fusion of a foreign protein or peptide to a second recombinant protein that has been shown to be stably expressed in plants can act to stabilize the target protein or peptide [24]. The fusion of antigen to the *Vibrio cholerae* toxin B subunit or the *E. coli* heat labile enterotoxin B subunit has contributed to the improvement of the humoral and cellular response due to adjuvant properties [46]. An alternative to this is the use of Hsp90 as a safer adjuvant. It has been demonstrated that LiHsp83, a member of the Hsp90 family, is a good candidate to carry antigens and develop an adjuvant-free vaccine [13]. Although future researches are necessary to understand the specific role of the pHsp90 as adjuvant, *e.g.* to evaluate whether these proteins are able to active macrophages and dendritic cells, the immunostimulatory properties of rAtHsp90 and rNbHsp90 observed here together with the high level of expression still under normal conditions, support the idea that these proteins could be excellent carriers to interesting vaccine antigens and peptides expressed in plants.

## Materials and Methods

### Animals

Female BALB/c (H-2d), C3H/HeN and C3H/HeJ mice bred and housed at the animal facilities of Biotechnology Research Institute (IIB), National University of General San Martin (UNSAM), Buenos Aires, Argentina, and used at the age of 8 to 10 weeks. All procedures requiring animals were performed in agreement with institutional guidelines and were approved by the Independent Ethics Committee for the Care and Use of Experimental Animals of National University of General San Martin (C.I.C.U.A.E., IIB-UNSAM), and approved and conducted in accordance with the guidelines established by the National University of General San Martin (SC055) and the National Research Agency (PICT 691).

### Plant material and heat-stress treatment

*Nicotiana benthamiana* plants were grown in growth chambers at 23°C under a 16 h photoperiod. Heat shock was administered by exposure of 3-week-old-seedlings to 42°C for 2 h. Plants were immediately harvested after the end of the stress period and stored at -80°C until their use for RNA isolation.

### Plasmid construction

Total RNA from frozen *N. benthamiana* tissue was obtained by grinding it in liquid nitrogen and then homogenizing it in Trizol (Invitrogen) reagent, following the manufacturer's protocol. Purified RNA was resuspended in DEPC-treated water and stored at −80°C. The concentration and purity of the RNA preparation was determined by measuring the absorbance at 260 and 280 nm. RNA integrity was analyzed on a 1% native agarose gel in 1X TBE buffer. cDNA synthesis was carried out using Super Script III (Invitrogen) and oligo dT_20_ (Invitrogen) under the manufacturer's conditions. This cDNA was used as template for PCR reactions for gene cloning and expression analysis. The forward primer used was 5′ - GGA TCC ATG GCG GAS GCA GAR ACS TTT GCW TTY CAA GC - 3′ and the reverse primer was 5′- AA CTG CAG TTA GTC YAC TTC CTC CAT CTT GCT ACC TTC AGC ATC - 3′. In addition, two restriction sites, *Bam*HI and *Pst*I (underlined), were added into the N-terminus and C-terminus, respectively. PCR conditions were: 94°C for 4 min, 25 cycles of 94°C for 25 s, 52°C for 30 s, 72°C for 1 min, and final extension 72°C for 7 min. The amplified product was ligated into the pGEM-T vector (Promega) and sequenced to confirm the presence of the sequence of the NbHsp90.3 mature protein. The recombinant plasmid was digested with *Bam*HI and *Pst*I and the insert was directionally cloned into a 6xHis-tagged pRSET-A expression vector resulting in the pRSET-A-NbHsp90.3 plasmid.

The construction of the plasmid pRSET-A expressing the mature protein of *A. thaliana* Hsp81.2 (AtHsp81.2) was carried out by PCR amplification of the plasmid RAFL 09-06-O18 containing the full-length AtHsp81.2 isoform (provided by RIKEN BRC) [47], [48] using the primers described above. The PCR product was ligated into the pGEM-T vector (Promega) and sequenced to confirm the presence of the insert. The recombinant plasmid was digested with *Bam*HI and *Pst*I and the insert was directionally subcloned into a 6xHis-tagged pRSET-A expression vector resulting in the pRSET-A-AtHsp81.2 plasmid.

### Expression and purification of recombinant proteins

*Escherichia coli* Rosetta (DE3) competent cells were transformed by pRSET-A-NbHsp90.3 and pRSET-A-AtHsp81.2. One colony was inoculated into Luria–Bertani (LB) medium supplemented with 100 µg/ml ampicillin and 20 µg/ml choramphenicol and incubated overnight at 37°C. Cultures were diluted with 200 ml LB (100-fold) medium containing 100 µg/ml ampicillin and 20 µg/ml choramphenicol and further incubated at 37°C up to a cell density of 0.5 (absorbance at 600 nm). Protein expression was induced by isopropyl-β-D-thiogalactoside (IPTG) to a final concentration of 1 mM for 2 h. Cells were harvested by centrifugation and stored at −20°C until use.

All purification procedures were carried out at 4°C. *E. coli* pREST-A-AtHsp81.2 and pRESET-A-NbHsp90.3 from 200 ml of culture were suspended in pre-cooled lysis buffer (300 mM NaCl, 50 mM HEPES pH 8, 10 mM imidazole) and phenyl-methyl-sulfonyl fluoride (PMSF) 1 mM was added. Cells were disrupted by sonication on ice (3 s pulse with 3 s intervals for 15 min) using 130 watt ultrasonic processor (Vibra Cell™, Sonic). The supernatant was collected by centrifugation for 15 min at 13,200 g at 4°C. Soluble AtHsp81.2 and NbHsp90.3 was purified under non-denaturing conditions using a nitrilotracetic acid-Ni^2+^ column (Qiagen). The proteins from eluted fractions were separated by SDS-PAGE (12%) using the Mini-Protean system III (Bio-Rad). After electrophoresis, proteins were stained with Coomassie Brilliant Blue. PageRuler™ Prestained Protein Ladder (Fermentas) was used as molecular marker. Stained bands were excised and subjected to sequencing by MALDI-TOF-TOF spectrometer, Ultraflex II (Bruker), in the mass spectrometry facility CEQUIBIEM, Argentina.

### Western blot Analysis

For Western blot analysis, proteins from the eluted fraction or total soluble protein extracted from *N. benthamiana* and *A. thaliana* leaves were incubated at 100°C for 5 min in loading buffer, separated by SDS-PAGE (10% gel) and transferred onto nitrocellulose membranes (GE) using an Electro-transfer unit (Bio-Rad). The membranes were sequentially incubated with mouse polyclonal anti-AtHsp81.2 antibody (1∶500) and alkaline phosphatase conjugated goat anti-mouse IgG (1∶5,000; Sigma). After washing, the reaction was developed by the addition of nitroblue tetrazolium/5-bromo-4-chloro-3-indolyl phosphate (NBT/BCIP) substrate. PageRuler™ Prestained Protein Ladder (Fermentas) was used as molecular marker.

### Colorimetric determination of ATPase activity

The ATP hydrolysis assay procedure was based on that previously described [25], [49], [50]. AtHsp81.2 (22.5 µg) was added to 250 µl of assay buffer (10 mM Hepes pH 8, 100 mM KCl, 2 mM MgCl_2_ and 0.5 mM DTT) and incubated for 5 min at 37°C. The reaction was started by the addition of ATP at various concentrations (0–5 mM). On the day of use, the colored buffer was prepared as follows: 0.045% malachite green, 1.5% ammonium molybdate in 4N HCl in a ratio 1∶1 (v∶v) and mixed for 20 min at room temperature. Then, it was filtered and Tween 20 at a final concentration of 0.19% was added. Color development was started by the addition of 800 µl of colored buffer to the samples, incubation for 1 min and final addition of 100 µl of 34% sodium citrate; after this, the colored samples were read at 660 nm (Lambda 25 UV/VIS spectrometer, Perkin Elmer). A blank with all the reagents except the chaperones was used to account for intrinsic hydrolysis and was subtracted from the sample readings. The nmoles of Pi released were calculated by comparison to a standard curve determined for inorganic phosphate. To allow comparisons between screens performed at different times, a phosphate standard curve (using sodium phosphate) was generated each day. Specific activity was determined in units of nmoles of phosphate released per minute per milligram of rpHsp90.

### Proliferation assays and stimuli

Whole spleens cells were isolated and cultured in complete Roswell Park Memorial Institute (RPMI) medium (RPMI 1640 supplemented with L-glutamine, 20% fetal bovine serum (FBS), 1% antibiotic anti-mycotic solution and 5×10^−5^ M 2β-mercaptoethanol). Lysis of red blood cells was achieved by incubating cells in lysis buffer (150 mM NH_4_Cl, 10 mM CO_3_HK, 1 mM EDTA, pH 7.4) at 37°C for 7 min. Afterwards, splenocytes were washed twice with complete medium and 2.5×10^5^ cells per well were plated in a 96-well flat-bottom microtiter plate in the presence of 12, 25, 50, 100, 150 and 200 µg/ml recombinant AtHsp81.2 or NbHsp90.3, 50 µg/ml recombinant *Toxoplasma gondii* Hsp28 (TgHsp28), 25 µg/ml recombinant *Leishmania infantum* Hsp83 (LiHsp83), 5 µg/ml ConA (Sigma) or 10 µg/ml LPS (Sigma). The concentration of LPS was measured using the HEK-Blue™ LPS Detection Kit (InvivoGen). Before quantification of endotoxin contamination, the recombinant proteins were heat treated (100°C, 20 min). rAtHsp81.2, rNbHsp90.3, rLiHsp83 and rTgHsp28 presented LPS contamination between 1 and 5 ng/ml, a concentration well below what is needed for mitogen proliferation [51], [52]. However, when splenocytes were stimulated with the recombinant proteins, 2 µg/ml polymyxin B sulphate (PX; Sigma) was added to the culture to abolish any possibility of LPS induced proliferation. Splenocytes were incubated for 72 h at 37°C in 5% CO_2_. Twenty-four hours before the end of the incubation period, 1 µCi of [^3^H]-thymidine (20 Ci/mmol, Perkin Elmer) was added to each well. Cells were harvested onto glass fiber filters. Incorporated radioactivity was measured in a liquid scintillation Beta counter (Beckman).

### Flow cytometry analysis

Splenocytes, 2.5×10^5^ cells/well, were incubated for 72 h at 37°C in 5% CO_2_ in the presence of different stimuli. The concentrations of the different stimuli were: recombinant proteins (AtHsp81.2 and NbHsp90.3) 50 µg/ml, ConA 5 µg/ml or LPS 10 µg/ml. Cells were washed twice with flow buffer (PBS, 3% fetal bovine serum, 0.1% sodium azide, 10 mM HEPES) and 10^6^ cells were incubated with anti-Fc (obtained from a hybridoma) for 15 min. After washing with flow buffer the cells were incubated with phycoerythrin (PE)-conjugated anti-mouse CD19 (BD Pharmingen) and fluorescein isothiocyanate (FITC)–conjugated anti-mouse CD3 (BD Pharmingen) for 30 min at 4°C in darkness. The cells were then washed and fixed with 200 µl of 0.5% paraformaldehyde in PBS. Acquisition of cells was done using a FACScan flow cytometer (BD Biosciences) appropriately set-up for two- color fluorometry. Dead cells were excluded on the basis of forward- and side-cell scatter. Background values obtained with fluorochrome conjugate isotype controls (PE-rat IgG2a, BD Pharmingen and FITC-rat IgG2b, e-Bioscience) were subtracted. Results were analyzed using WinMDI 2.9 software.

### Fractionation of B- and T-cells

Whole cells were isolated in complete Roswell Park Memorial Institute (described above). Red blood cells were lysed by incubating them in lysis buffer (150 mM NH_4_Cl, 10 mM CO_3_HK, 1 mM EDTA, pH 7.4) at 37°C for 7 min. Afterwards, splenocytes were washed twice with flow buffer and centrifuged for 10 min at 250 g at 4°C and counted. Fifteen million cells were incubated in flow buffer without sodium azide with 1 µl of phycoerythrin (PE)-conjugated anti-mouse CD19 (BD Biosciences) per million of cells and 1 µl of FITC–conjugated anti-mouse CD3 (BD Biosciences) per million of cells for 30 min at 4°C in darkness. After washing twice with flow buffer, the cells were resuspended in the same buffer. Cells were sorted using a BD FACSAria II cell sorter (BD Biosciences). Cells were analyzed using the Winmdi software. After sorting, 200 µl of cells (1×10^5^) per well were plated in a 96-well flat-bottom microtiter plate in the presence of medium or 50 µg/ml recombinant AtHsp81.2 or NbHsp90.3. Splenocytes were incubated for 72 h at 37°C in 5% CO_2_. Twenty-four hours before the end of the incubation period, 1 µCi of [^3^H]thymidine (20 Ci/mmol, Perkin Elmer) was added to each well. Cells were harvested onto glass fiber filters. Incorporated radioactivity was measured in a liquid scintillation Beta counter (Beckman).

### Indirect immunofluorescence assay

Sterile medium (1 ml) with 2.5×10^5^ of spleen cells and 50 µg/ml of rAtHsp81.2 was plated on a sterile coverslip at room temperature for 30 min. Cells were fixed with 4% (v/v) paraformaldehyde, washed twice with PBS and permeabilized with 0.2% Triton X-100 in PBS for 10 min. After washing with PBS, cells were blocked with 10% (w/v) bovine serum albumin for 30 min and incubated with the appropriate dilution of each primary antibody for 1 h, using mouse anti-6His mAb (Qiagen) (1∶500). Following incubation, cells were washed three times with PBS, and then incubated with the corresponding secondary antibodies Alexa Fluor 594 goat anti-mouse IgG (1∶4000) (Invitrogen, red color) or Alexa Fluor 488 goat anti-mouse IgG (1∶4000) (Invitrogen, green color) for 1 h. Following incubation, cells were washed three times with PBS, and then incubated with the corresponding tertiary antibodies mouse phycoerythrin (PE)-conjugated anti-mouse CD19 mAb (1∶250) (BD, red color) or mouse FITC-conjugated anti-mouse CD3 mAb (1∶250) (BD, green color). Following antibody labeling, coverslips were incubated in 2.8 mM 40,6-diamidino-2-phenylindole (DAPI; Molecular Probes) for 10 min. Cover slips were washed three times and mounted in Fluorescent Mounting Medium (Dako) and viewed using a Nikon Model Eclipse E600 (magnification 100X, numerical aperture 1.40 at 24°C) equipped with a 100 W Hg-vapor lamp and epifluorescence filter sets and a Nikon DS-Qi 1 Mc monochromatic camera. DAPI staining reveals the location of the nucleus (blue color). Green, red and blue fluorescence were recorded in separate channels and the images merged using Image-Pro Plus 4.5 (Media Cybernetics, Inc.).

### Sequence analysis of Hsp90 proteins

The sequences of Hsp90 proteins from *N. benthamiana* (accession N° GQ_845021), *A. thaliana* (accession N° NM_124985), *L. infantum* (accession N° XM_001468081), *S. cerevisia* (accession N° NM_001182692), *H. sapiens* (accession N° NM_007355) and *E. coli* (accession N° NP_415006) were downloaded from GenBank (www.ncbi.nlm.nih.gov/genbank/). Multiple sequence alignment was performed using CLUSTALX [53] as implemented in BioEdit (Tom Hall, Isis Pharmaceuticals, Inc.).

### Statistical analysis

Statistical analysis was carried out with the Prism 5.0 Software (GraphPad, San Diego, CA) using one-way and two-way analysis of variance (ANOVA). Values of p<0.05 were considered significant.

## Supporting Information

Figure S1 **Polymyxin B did not inhibit rpHsp90-induced proliferative responses.** The splenocytes were incubated for 72 h at 37°C in 5% CO_2_. The concentrations of the different stimuli were: LPS 10 µg/ml, rAtHsp81.2 50 µg/ml and rNbHsp90.3 50 µg/ml with and without polymyxin B (PX) at 2 µg/ml. rAtHsp81.2: recombinant heat shock protein 81.2 from *Arabidopsis thaliana*; rNbHsp90.3: recombinant heat shock protein 90.3 from *Nicotiana benthamiana*. a: indicates the statistically significant differences between the different treatments (LPS vs. LPS + PX, p<0.001); b: indicates the statistically significant differences between stimuli and the negative controls (LPS vs. medium and LPS + PX; and rAtHsp81.2 vs. medium and LPS + PX, p<0.001; and rNbHsp90.3 vs. medium and LPS + PX, p<0.01). Values represent the mean counts per minute and standard deviations of triplicate samples from three mice and are representative of two experiments. Statistical analysis was performed by two-way analysis of variance (ANOVA) using the Bonferroni's Post-Test and one-way analysis of variance (ANOVA) using the Bonferroni's Multiple Comparison Test.
(TIF)

Figure S2 **Localization of the rAtHsp81.2 at the plasma membrane from spleen cells of C3H/HeJ mice.** Spleen cells were stimulated with rAtHsp81.2 or incubated with medium for 30 min. (A) CD3^+^ cells were incubated with mouse anti-6His mAb as primary antibody, Alexa Fluor 594 goat anti-mouse IgG (red color) as secondary antibody and fluorescein isothiocyanate (FITC)-conjugated anti-mouse CD3 mAb (BD, green color). Nuclei were stained with DAPI. The three images were merged (CD3 + rAtHsp81.2 + nucleus). Scale bar represents 1 µm. This image is representative of a larger field of view, and data are from a representative experiment performed three times. Green, red and blue fluorescence were recorded separately, and the images were merged using image-pro plus 4.5. (B) CD19^+^ cells were incubated with mouse anti-6His mAb as primary antibody, Alexa Fluor 488 goat anti-mouse IgG (green color) as secondary antibody and phycoerythrin (PE)-conjugated anti-mouse CD19 mAb (BD, red color) as tertiary antibody. Nuclei were stained with DAPI. The three images were merged (CD19 + rAtHsp81.2 + nucleus).
(TIF)

Figure S3 **AtHsp81.2 co-localizes with CD19 cells but not with CD3 cells.** Spleen cells were incubated with rAtHsp81.2. (A) CD3^+^ cells were incubated with mouse anti-6His mAb as primary antibody, Alexa Fluor 594 goat anti-mouse IgG (red color) as secondary antibody and fluorescein isothiocyanate (FITC)-conjugated anti-mouse CD3 mAb (BD, green color). Nuclei were stained with DAPI. (B) CD19^+^ cells were incubated with mouse anti-6His mAb as primary antibody, Alexa Fluor 488 goat anti-mouse IgG (green color) as secondary antibody and phycoerythrin (PE)-conjugated anti-mouse CD19 mAb (BD, red color) as tertiary antibody. Nuclei were stained with DAPI. (A and B) Scale bar represents 1 µm. These images are representative of a larger field of view, and data are from a representative experiment performed three times. PC: phase contrast. Green, red and blue fluorescence were recorded separately, and the images were merged using image-pro plus 4.5.
(TIF)
