# Supplementary figures and images for: Plant Hsp90 Proteins Interact with B-Cells and Stimulate Their Proliferation
Source: PLoS One. 2011 Jun 20;6(6):e21231. doi: 10.1371/journal.pone.0021231 (PMC3118808; doi:10.1371/journal.pone.0021231)

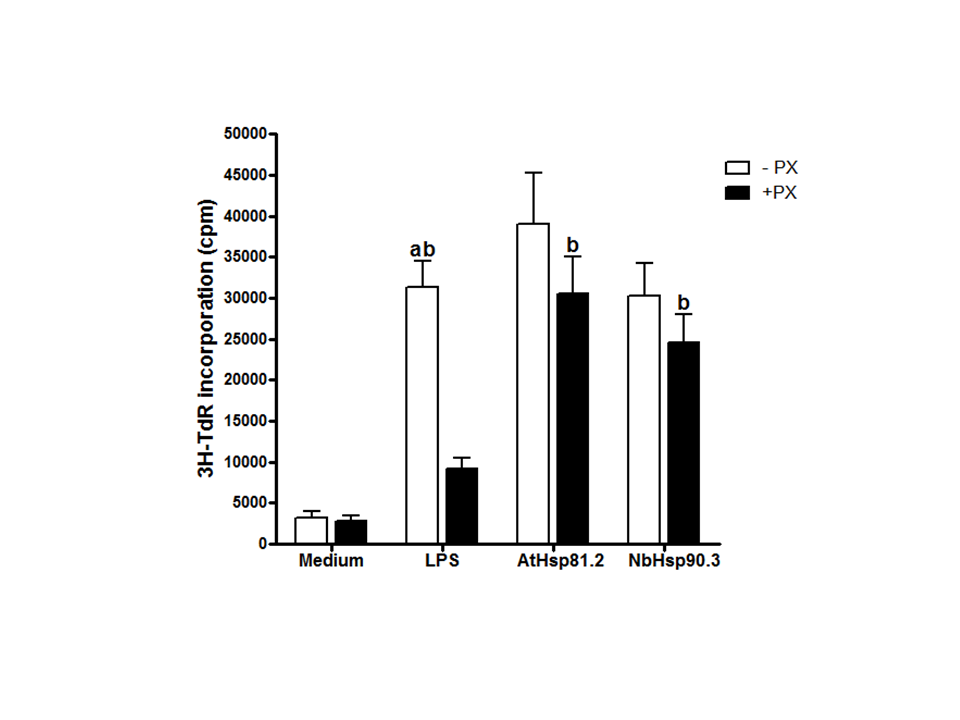

Supplement: Figure S1 — Polymyxin B did not inhibit rpHsp90-induced proliferative responses. The splenocytes were incubated for 72 h at 37°C in 5% CO2. The concentrations of the different stimuli were: LPS 10 µg/ml, rAtHsp81.2 50 µg/ml and rNbHsp90.3 50 µg/ml with and without polymyxin B (PX) at 2 µg/ml. rAtHsp81.2: recombinant heat shock protein 81.2 from Arabidopsis thaliana; rNbHsp90.3: recombinant heat shock protein 90.3 from Nicotiana benthamiana. a: indicates the statistically significant differences between the different treatments (LPS vs. LPS + PX, p<0.001); b: indicates the statistically significant differences between stimuli and the negative controls (LPS vs. medium and LPS + PX; and rAtHsp81.2 vs. medium and LPS + PX, p<0.001; and rNbHsp90.3 vs. medium and LPS + PX, p<0.01). Values represent the mean counts per minute and standard deviations of triplicate samples from three mice and are representative of two experiments. Statistical analysis was performed by two-way analysis of variance (ANOVA) using the Bonferroni's Post-Test and one-way analysis of variance (ANOVA) using the Bonferroni's Multiple Comparison Test. (TIF) [file pone.0021231.s001.tif]

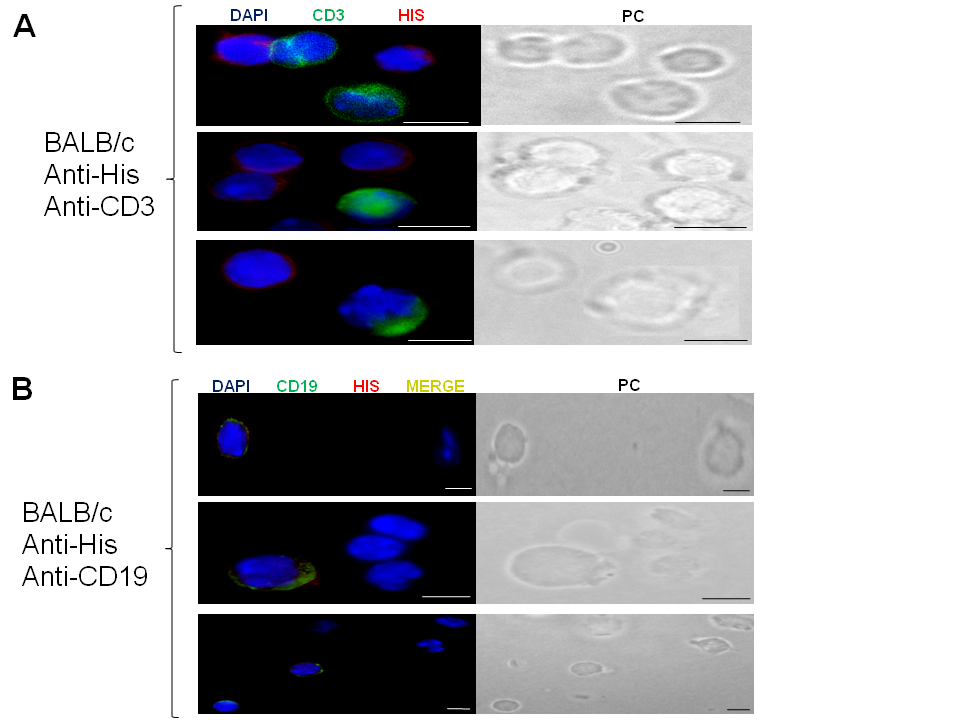

Supplement: Figure S2 — Localization of the rAtHsp81.2 at the plasma membrane from spleen cells of C3H/HeJ mice. Spleen cells were stimulated with rAtHsp81.2 or incubated with medium for 30 min. (A) CD3+ cells were incubated with mouse anti-6His mAb as primary antibody, Alexa Fluor 594 goat anti-mouse IgG (red color) as secondary antibody and fluorescein isothiocyanate (FITC)-conjugated anti-mouse CD3 mAb (BD, green color). Nuclei were stained with DAPI. The three images were merged (CD3 + rAtHsp81.2 + nucleus). Scale bar represents 1 µm. This image is representative of a larger field of view, and data are from a representative experiment performed three times. Green, red and blue fluorescence were recorded separately, and the images were merged using image-pro plus 4.5. (B) CD19+ cells were incubated with mouse anti-6His mAb as primary antibody, Alexa Fluor 488 goat anti-mouse IgG (green color) as secondary antibody and phycoerythrin (PE)-conjugated anti-mouse CD19 mAb (BD, red color) as tertiary antibody. Nuclei were stained with DAPI. The three images were merged (CD19 + rAtHsp81.2 + nucleus). (TIF) [file pone.0021231.s002.tif]

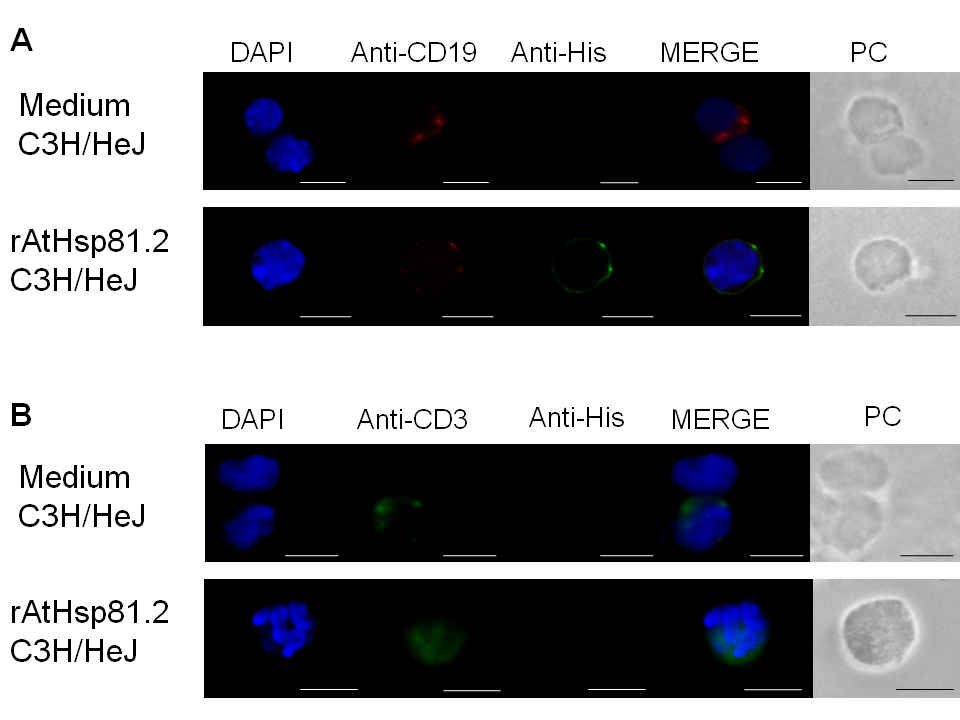

Supplement: Figure S3 — AtHsp81.2 co-localizes with CD19 cells but not with CD3 cells. Spleen cells were incubated with rAtHsp81.2. (A) CD3+ cells were incubated with mouse anti-6His mAb as primary antibody, Alexa Fluor 594 goat anti-mouse IgG (red color) as secondary antibody and fluorescein isothiocyanate (FITC)-conjugated anti-mouse CD3 mAb (BD, green color). Nuclei were stained with DAPI. (B) CD19+ cells were incubated with mouse anti-6His mAb as primary antibody, Alexa Fluor 488 goat anti-mouse IgG (green color) as secondary antibody and phycoerythrin (PE)-conjugated anti-mouse CD19 mAb (BD, red color) as tertiary antibody. Nuclei were stained with DAPI. (A and B) Scale bar represents 1 µm. These images are representative of a larger field of view, and data are from a representative experiment performed three times. PC: phase contrast. Green, red and blue fluorescence were recorded separately, and the images were merged using image-pro plus 4.5. (TIF) [file pone.0021231.s003.tif]
